# Analysis of DRD2 Gene Polymorphism in the Context of Personality Traits in a Group of Athletes

**DOI:** 10.3390/genes12081219

**Published:** 2021-08-06

**Authors:** Krzysztof Chmielowiec, Monika Michałowska-Sawczyn, Jolanta Masiak, Jolanta Chmielowiec, Grzegorz Trybek, Marta Niewczas, Wojciech Czarny, Paweł Cieszczyk, Myosotis Massidda, Patrizia Proia, Anna Grzywacz

**Affiliations:** 1 Department of Hygiene and Epidemiology, Collegium Medicum, University of Zielona Góra, 65-046 Zielona Góra, Poland; chmiele@vp.pl (K.C.); chmiele1@o2.pl (J.C.); 2 Faculty of Physical Culture, Gdańsk University of Physical Education and Sport, 80-336 Gdańsk, Poland; monikamichalowska@op.pl (M.M.-S.); cieszczyk@poczta.onet.pl (P.C.); 3 II Department of Psychiatry and Psychiatric Rehabilitation, Medical University of Lublin, 20-059 Lublin, Poland; jolanta.masiak@umlub.pl; 4 Department of Oral Surgery, Pomeranian Medical University, 70-111 Szczecin, Poland; g.trybek@gmail.com; 5 Faculty of Physical Education, University of Rzeszów, 35-959 Rzeszów, Poland; martaniewczas@wp.pl (M.N.); wojciechczarny@wp.pl (W.C.); 6 Department of Life and Environmental Sciences, University of Cagliari, 72-09124 Cagliari, Italy; myosotis.massidda@gmail.com; 7 Sport and Exercise Sciences Research Unit, Department of Psychological, Pedagogical and Educational Sciences, University of Palermo, 90133 Palermo, Italy; patrizia.proia@unipa.it; 8 Independent Laboratory of Health Promotion, Pomeranian Medical University in Szczecin, 70-204 Szczecin, Poland

**Keywords:** genetics, personality traits, dopamine, D2 receptor, athletes

## Abstract

The presented study showed the relationship between dopamine receptor gene polymorphism and personality traits in athletes training in martial arts. Behavioral modulation resulting from a balance of the neurotransmitters dopamine and norepinephrine to inactivation of the dorsolateral prefrontal cortex and dysregulation of various pathways involved in attention and impulse control processes; Methods: The study was conducted among martial arts athletes. The study group included 258 volunteers and 284 controls. The genetic test was performed using the real-time PCR method; psychological tests were performed using standardized TCI questionnaires. All analyses were performed using STATISTICA 13. Results: Interaction between martial arts and DRD2 rs1799732 (manual) G/-(VIC/FAM)-ins/del and RD- Harm avoidance and Reward Dependence scale were demonstrated. In athletes, a lower Reward Dependence scale score was associated with the DRD2 rs1799732 (manual)-/-polymorphism compared to the control group. Conclusions: It seems justified to study not only genetic aspects related to brain transmission dopamine in martial arts athletes. In the studied athletes, the features related to reward addiction and harm avoidance are particularly important in connection with the dopaminergic reward system in the brain.

## 1. Introduction

Possible links between a tendency to participate in high-risk activities and genetic markers have been considered by geneticists. A probable connection between polymorphisms of the D2 subtype dopamine 2 receptor, a G protein-coupled receptor, the inhibitor of adenyl cyclase, and risk-taking, novelty-seeking behavior in humans and other living organisms seem to show a link from a perspective of teleology [1,2,3,4]. The research concerning the group of skiers and snowboarders conducted by Thomson and associates [3] is especially interesting. Dopamine is treated as the neurotransmitter highly conditioning “action”, addiction and substance abuse. Extreme sport participants and their risk-taking behaviors are conditioned with adrenaline /dopamine/ endorphin surges. The same inflow of dopamine is observed in gambling and risk-heavy professions such as financial trading, which strongly attracts participants as their chosen “edge work” [5]. 

Moreover, numerous mental functions and behaviors are conditioned with dopamine and its role in neurotransmission. 

The *DRD2* gene polymorphisms are evidently strongly grounded in the neurobiology and functioning of the human brain, its neurotransmitters, and receptors. Moreover, in the literature on the subject, a relationship between receptor polymorphism (DA2) and arterial hypertension has been shown through increased secretion of catecholamine release. Rosmond et al. [6] report the association in exon 6 of the dopamine D2 gene receptor (DRD2) with heart disease. It is worth mentioning here the role of dopamine considered by psychologists and psychiatrists as “the hormone of motivation to act and searching for new emotions” [7]. In this aspect, dopamine has a fundamental influence on making so-called “risky decisions.” Genes coding receptors and transporter of this neurotransmitter will be considered in case of sport predispositions. It is connected with the brain pathway influencing the pleasure and satisfaction feeling. We can also mention here the so-called “mesolimbic reward pathway” that mediates in reward psychopharmacology, induced among the others with physical effort. In such a case, no matter the situation, brain area in the sphere of ventral tegmental area (the place of dopaminergic pathway neurons location) and functionally connected nucleus accumbens are described as “pleasure center” and the dopamine itself “neurotransmitter of pleasure.” In this context, it can be presumed that the system described above can be one of the key determinants for deciding and following the continuation of sports training [1,4,7].

Another argument indicating dopamine significance in the area of sports achievements is its influence on the process of vision. In the case of sport, the fight is one of the key determinants of potential success since dopamine plays the role of paracrine neurotransmitter that is “chemical analogs of light” in the retina, located on photoreceptors cells receptors of D2 and D4 control light-dependent processes such as melatonin biosynthesis, groups of opsin protein expression in cones or the level of internal photoreceptor cAMP [1,4,7].

Dopamine secretion and metabolism also show a strong influence on personality traits [8]. Because of the facts mentioned above, several researchers considered personality traits in connection with dopamine-related genes. Especially strong interest is observed regarding the 48 bps variable number tandem repeat (VNTR) polymorphism in exon 3 of the dopamine D4 receptor (*DRD4*) gene influencing novelty seeking (NS) [9]. Nonetheless, only a restricted number of studies have considered other dopamine receptor genes, such as dopamine D2 receptor (*DRD2*) and dopamine D3 receptor (*DRD3*) genes. Both of them, to some extent, resemble DRD4, the DRD2 structurally, and DRD3 with its pharmacologic profile. Moreover, the volume of DRD4 binding affinity to a dopamine agonist is extremely low compared to DRD2 [10]. Hence, the *DRD2* and *DRD3* genes can also be treated as a candidate for personality-related genes.

The modulation of behavior resulting from the dopamine and norepinephrine neurotransmitter surge behaves in reverse to under-activation in the dorsolateral prefrontal cortex and dysregulation of various pathways engaged in the attention and impulse control processes [11,12]. Cloninger’s work on personalities [13] concerning four dimensions of human behavior (harm avoidance, reward dependence, novelty seeking, and perseverance) was extremely helpful for evaluating the personality structures of extreme sports participants.

In athletic studies, genetic studies related to the characteristics of the temperament, along with medical examinations and other physiological and biochemical measurements, would enhance the picture of the possibilities and successes that an individual can achieve. This could have potential implications for the protection of both mental and physical health.

## 2. Materials and Methods

### 2.1. Research Group and Subject Recruitment—Research Course 

The study was conducted among martial arts athletes. The study group included 258 volunteers who gave their written consent to participate in the study. The volunteers were somatically healthy, a history of addiction and psychosis was excluded. The group consisted of men aged 26.02 ± 8.30 years. 

Controls included 284 unrelated, healthy (non-dependent and non-psychosis) Polish male volunteers aged 22.89 ± 4.77. All athletes and controls were Caucasian to reduce the possibility of racial gene skewing and to overcome any potential problems due to population stratification.

The study was conducted in accordance with the Declaration of Helsinki principles and approved by the Ethics Committee. All subjects provided signed informed consent for participating in the research. There was no financial or other compensation for being part of the sample of the study. The assessment process took place in a single session lasting about 100 min. Psychologists and psychiatrists collected data for the semi-structured interview with high experience in the treatment of athletes’ psychological tests. Additionally, the interviewed group could be helped by an assistant present in a room, who also checked the completeness of handled tests. All the procedures allowing comfort and concentration were accomplished. Psychology specialists accomplished tests interpretation. 

### 2.2. Genetic Tests 

The subjects and the controls were genotyped with TaqMan SNP Genotyping Assays. Genotyping process was accomplished with the Thermo Fisher Assay ID C_33641686_10 with context sequence [VIC/FAM] GTACCTCCTCGGCGATCCCCGGCCT[G/−]GAACGGGTAGGAGGGGTTGGGGGAT. DNA was extracted from the buccal cells using the High Pure PCR Template Preparation Kit (Roche, Basel, Switzerland) according to the manufacturer’s instructions. The genotyping mixture (total volume: 5 μL) contained 2.5 μL of TaqPath ProAmp Master Mix (ThermoFisher Scientific, Dreieich, Germany), 0.25 μL of assay mix (10×), and 1 μL of distilled water with 1.25 μL of genomic DNA (10 ng/μL) per reaction. The thermal cycling conditions included a pre-read at 60 °C for 30 s, an initial denaturation at 95 °C for 5 min, followed by 40 cycles of denaturation at 95 °C for 5 s and annealing/extension at 60 °C for 30 s, finished by a post-read at 60 °C for 30 s. Genotyping reaction was performed on the CFX Connect Real-Time PCR Detection System (BioRad, Hercules, CA, USA).

### 2.3. Psychological Tests 

Temperament and Character Inventory-Revised (TCI-R) is a self-report questionnaire developed to determine personality traits with the usage of 240 items based on Cloninger’s multidimensional model and structured into seven factors [four for temperament (novelty seeking, harm avoidance, reward dependence, and persistence), and three for character (self-directedness, cooperation, and self-transcendence)]. The psychometrical Polish adaptation of the tool obtained adequate properties [13,14].

### 2.4. Statistical Analysis

The DRD2 rs1799732 genotypes distribution was tested according to Hardy–Weinberg equilibrium (HWE) with the HWE software https://wpcalc.com/en/equilibrium-hardy-weinberg/ (accessed on 3 June 2021). 

The analyzed variables did not have a normal distribution. Results from the U Mann-Whitney test were used to determine the difference in analyzed traits of novelty seeking, harm avoidance, reward dependence, self-directedness, cooperation, and self-transcendence.

Not all assumptions required for the ANOVA analysis were met. The assumption about the normal distribution was not fulfilled for all dependent variables, but the variance was the same (Levene test *p* > 0.05). Because the number of subjects in groups was also large, it was therefore decided to use multivariate analysis 2 × 3 factorial ANOVA. The test was used to show an association between novelty seeking, harm avoidance, reward dependence, cooperation, self-transcendence results, and the martial arts and control group, and the *DRD2* rs1799732 polymorphism (personality traits × control and martial arts subjects × genetic feature).

The frequencies of genotypes and alleles of the *DRD2* rs1799732 polymorphism in an analyzed group were compared by the chi-square test. All analyses were performed using STATISTICA 13 (Tibco Software Inc., Palo Alto, CA, USA) for Windows (Microsoft Corporation, Redmond, WA, USA).

## 3. Results

The frequency distributions accorded with the HWE. There was a statistical difference between martial arts subjects and control subjects (Table 1).

The *DRD2* rs1799732 genotypes and alleles frequencies in the studied sample do not differ in analyzed groups subjects (Table 2).

The means and standard deviations for novelty seeking, harm avoidance, reward dependence, self-directedness, cooperation, self-transcendence in the group of martial arts subjects and control subjects are presented in Table 3. Compared to the controls, the case group subjects had significantly higher scores on self-directedness (M 26.72 vs. M 23.67, *p* < 0.0001). Lower scores on the scales of harm avoidance (M 9.71 vs. M 11.35, *p* < 0.0001) were noticed (Table 3).

### 3.1. Harm Avoidance and DRD2 rs1799732 

The result of the 2 × 3 factorial ANOVA was found for the combined factor *DRD2* rs1799732 genotype z martial arts/control (F_2536_ = 4.25, *p =* 0.0147, η^2^ = 0.016) (Table 4). Power calculation—our sample had more than 74% power to detect the combined factor martial arts/control × *DRD2* rs1799732 and their interaction effect (about 2% of the phenotype variance).

### 3.2. Reward Dependence and DRD2 rs1799732

**Figure 1:**
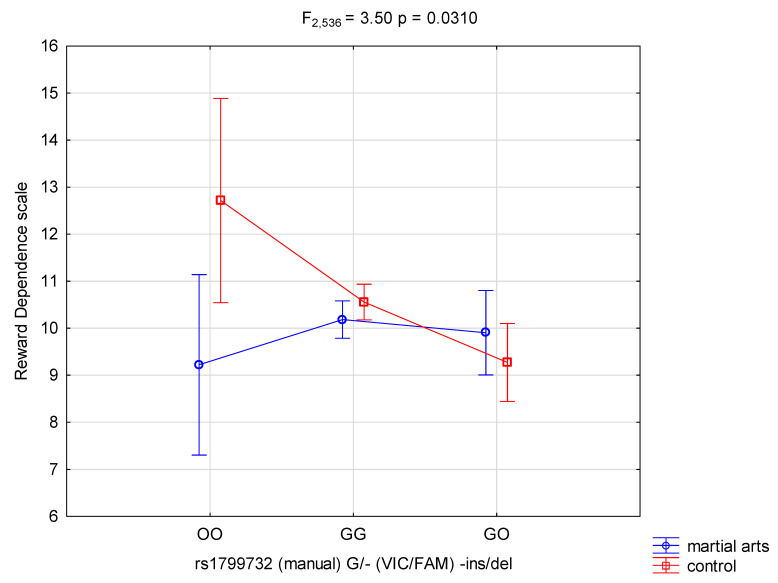
Interaction between martial arts/-control and *DRD2* rs1799732 (manual) G/-(VIC/FAM)-ins/del and RD-Reward Dependence scale.

The results of 2 × 3 factorial ANOVA of martial arts subjects and control subjects was found for reward dependence (F_1536_ = 3.95, *p =* 0.0472, η^2^ = 0.007) and the *DRD2* rs1799732 genotype was found for reward dependence (F_2536_ = 3.10, *p =* 0.0457, η^2^ = 0.011) (Table 4). Power calculation—our sample had 51% power to detect in martial arts and control subjects the effects of the studied reward dependence and their interaction effect (about 1% of the phenotype variance) and more than 60% power to detect the *DRD2* rs1799732 genotype effects of the studied reward dependence and their interaction effect (about 1% of the phenotype variance). We also noticed a statistically significant effect of combined factor *DRD2* rs1799732 genotype of martial arts/control (F_2536_ = 3.50, *p =* 0.0310, η^2^ = 0.013) (Table 4, Figure 1). Power calculation—our sample had more than 65% power to detect the combined factor of martial arts/control × *DRD2* rs1799732 and their interaction effect (about 1% of the phenotype variance).

### 3.3. Self-Directedness and DRD2 rs1799732

The results of 2 × 3 factorial ANOVA of martial arts subjects and control subjects was found for Self-directedness (F_1536_ = 4.42, *p =* 0.0358, η^2^ = 0.008) (Table 4). Power calculation —our sample had 56% power to detect in martial arts subjects and control subjects the effects of the self-directedness and their interaction effect (about 1% of the phenotype variance).

### 3.4. Cooperation and DRD2 rs1799732

The results of 2 × 3 factorial ANOVA of the *DRD2* rs1799732 genotype was found for cooperation (F_2536_ = 3.94, *p =* 0.0201, η^2^ = 0.014) (Table 4). Power calculation—our sample had 71% power to detect in the *DRD2* rs1799732 genotype effects of the studied cooperation and their interaction effect (about 1% of the phenotype variance).

### 3.5. Self-Transcendence and DRD2 rs1799732

The results of 2 × 3 factorial ANOVA of the *DRD2* rs1799732 genotype was found for self-transcendence (F_2536_ = 3.793, *p =* 0.0231, η^2^ = 0.013) (Table 4). Power calculation—our sample had 69% power to detect in the *DRD2* rs1799732 genotype effects of the studied self-transcendence and their interaction effect (about 1% of the phenotype variance).

## 4. Discussion

Testing the athletes′ personalities in the context of their success has been done for a long time. Beckmann and Kazen [15] observed that controlled type sports athletes whose demands connected with energy regulation were high (long-distance runners and rowers) are predisposed to suffer from failure-related state orientation or the shortage of motivation. The tendency can be combined with reward dependence traits within our research group. In other studies, it was found that people with higher novelty-seeking easily lose determination in the situation that does not meet their needs [16,17]. Morgan [18] noticed that male distance runners claimed lower stress, depression, anger, and tiredness compared to an average person. Egloff and Gruhn [19] suggested that in the case of endurance athletes, extraversion and sociability are the traits that strongly influence the choice of sport. Extraversion is characterized by sociability, controlled impulsiveness, and optimism [20]. Bäckmand et al. [21] insisted that endurance sport athletes had lower neuroticism scores than other sports athletes.

It is evident that the development of molecular biology methods has allowed searching for biological associations in this range. As it was justified in the introduction, dopamine can play a key role in sport motivation and determination. Individual genetic differences will be demonstrated among the others with the modulatory influence of the neurotransmitters’ system on the expression of the determined personality traits. An example can be novelty seeking (represented among the others with “hunger” for strong sensations) resulting from dopaminergic system functioning, more precisely, dopamine deficiency. Evidence for the legitimacy of gene coding dopamine receptor choice for our research can be found as early as in the analysis concerning neuroimaging. In the area of imaging-based analysis, an association between the density of the *DRD2* and the personality trait of novelty seeking (NS) was noticed [22]. Farde et al. [23] noticed a significant correlation between *DRD2* amount in the brain and a detached personality. What was also presented by Breier et al. [24] TaqI polymorphism in the 3′ region as not functionally active was one of the first areas of attention in molecular genetic study connected with personality traits. The association between the TaqI A1 allele and NS has been mainly investigated [25]. Several functional polymorphisms have been studied in the *DRD2* gene. The Ser311Cys polymorphism is a missense mutation located in exon 7, the putative third cytoplasmic loop. The Cys allele was shown to be less effective than the Ser allele in inhibiting cAMP synthesis [26], indicating a functional deficit of the Cys allele. However, no association was observed between the polymorphism and personality traits in Gebhardt et al. [27].

The research of Hibino from 2006 [28] investigated the association of polymorphisms in the three dopamine-related genes. We have chosen three genes connected with dopamine for this research—genes of dopamine receptors *DRD2*, *DRD3*, and *TH*. Analysis was conducted in connection with personality traits. Additionally, the authors analyzed epistasis among the genes and the analysis based on each gene. As a result, in the analysis based on each gene, trends for association were observed between State Anxiety and the *DRD2*-141C Ins/Del polymorphism and between Trait Anxiety and the *DRD2* Ser311Cys or *TH* PstI site polymorphism. In epistatic analysis, a trend for interaction was observed on the Neuroticism and Trait Anxiety scores between the *DRD2*-141C Ins/Del and *TH* Val81Met polymorphisms. Nonetheless, the research conclusion did not provide evidence for the association between these dopamine-related genes, *DRD2*, DRD3, *TH*, and personality traits (significance was not observed after using the Bonferroni correction). However, we have to be careful with discussion in relation to our own research. Hence, Hibino’s research considered the Japanese population.

However, other researchers also looked for the association between the dopamine receptor gene and personality traits considering functional polymorphisms of this gene. Jonsson and others [29], in their association research, did not notice a connection between the Temperament and Character Inventory (TCI) [14] in Caucasians, even though they observed association with personality. They concluded that the role of these functional polymorphisms is slight in relation to personality traits. Significance with personality traits and *DRD3*, *DRD4* genes, and reward system was reported by Ebstein. However, in their research, the significance after usage of the Bonferroni correction was not observed.

## 5. Conclusions

It seems justified to study not only genetic aspects related to brain transmission in martial arts athletes. It is also important to define temperamental traits as an image of a biological predisposition to specific sports. The features related to Reward Dependence and Harm avoidance are of particular importance in connection with the dopaminergic reward system in the brain.

## Figures and Tables

**Table 1:** Hardy–Weinberg equilibrium of the *DRD2* rs1799732 (manual) G/−(VIC/FAM) -ins/del in the group of martial arts subjects and controls.

Group	*DRD2* rs1799732 (Manual) G/-(VIC/FAM)-ins/del				
		Observed (Expected)	Alleles Frequency	χ^2^	*p* Value
Martial arts *N* = 258	GG	208 (202.37)	p allele freq (C) = 0.89 q allele freq (A) = 0.11	11.967	<0.0001
	OO	9 (3.37)			
	GO	41 (52.25)			
Controls *N* = 284	GG	229 (225.38)	p allele freq (C ) = 0.89 q allele freq (A) = 0.11	4.87	0.027
	OO	7 (3.38)			
	GO	48 (55.23)			

*p*-statistical significance, χ^2^-Chi^2^ test result, *N*-number of subjects.

**Table 2:** Frequency of genotypes and alleles of the *DRD2* rs1799732 (manual) G/−(VIC/FAM)-ins/del polymorphism group of martial arts subjects and controls.

Group	*DRD2* rs1799732 (Manual) G/-(VIC/FAM)-ins/del Genotypes Alleles				
	GG N(%)	OO N(%)	GO N(%)	G N(%) 516/568	O N(%)
Martial arts *N* = 258	208 (0.81)	9 (0.03)	41 (0.16)	457 (0.89)	59 (0.11)
Controls *N* = 284	229 (0.81)	7 (0.02)	48 (0.17)	506 (0.89)	62 (0.11)
χ^2^ *p* value	0.564 0.754			0.073 0.786	

*p*-statistical significance, χ2-Chi^2^ test result, *N*-number of subjects.

**Table 3:** Analysis of novelty seeking, harm avoidance, reward dependence, self-directedness, cooperation, self-transcendence results in martial arts subjects and controls.

	Martial Arts (*N* = 258) M ± SD	Control (*N* = 284) M ± SD	U Mann-Whitney Z	*p* Value
Genotypes *DRD2* rs1799732 (%)	GG (0.81) OO (0.03) GO (0.16)	GG (0.81) OO (0.02) GO (0.17)		
Alleles *DRD2* rs1799732 (%)	G (0.89) O (0.11)	G (0.89) O (0.11)		
Novelty seeking	20.10 ± 4.79	20.27 ± 4.63	−0.266	0.7904
Harm avoidance	9.71 ± 4.83	11.35 ± 4.60	−4.163	0.00003
Reward dependence	10.10 ± 3.01	10.39 ± 2.90	−0.850	0.3952
Self-directedness	26.72 ± 4.32	23.67 ± 5.08	7.071	<0.0001
Cooperation	20.59 ± 4.54	19.98 ± 4.63	1.698	0.0895
Self-transcendence	6.95 ± 3.56	7.04 ± 3.53	−0.367	0.7139

M—mean, SD—standard deviation, U Mann–Whitney Z-test. Statistically significant between-group differences are marked in bold print. *DRD2* rs1799732 (manual) G/-(VIC/FAM)-ins/del polymorphism.

**Table 4:** The results of 2 × 3 factorial ANOVA for martial arts subjects and controls, incorporating novelty seeking, harm avoidance, reward dependence, self-directedness, cooperation, self-transcendence results, and *DRD2* rs1799732 (manual) G/-(VIC/FAM)-ins/del.

	*DRD2* rs1799732					2 × 3-Factor ANOVA				
	Martial Arts (N = 258) M ± SD	Control (N = 284) M ± SD	GG (N = 437) M ± SD	GO (N = 89) M ± SD	OO (N = 23) M ± SD	Full Model F (*p* Value)	Factor	F (*p* Value)	η^2^	Power (Alfa = 0.05)
Novelty seeking	20.10 ± 4.79	20.27 ± 4.64	20.17 ± 4.66	20.64 ± 4.88	18.06 ± 4.78	F_5536_ = 0.957 *p =* 0.4436 R^2^ = 0.009	intercept	F_1536_ = 2025.50 (*p* < 0.0001)	0.791	1.000
							Martial arts/control	F_1536_ = 0.22 (*p* < 0.6401)	0.0004	0.075
							*DRD2* rs1799732	F_2536_ = 1.92 (*p =* 0.1479)	0.007	0.398
							Martial arts/control × *DRD2* rs1799732	F_2536_ = 0.287 (*p =* 0.7500)	0.001	0.095
Harm avoidance	9.71 ± 4.82	11.36 ± 4.60	10.47 ± 4.82	10.90 ± 4.70	11.56 ± 3.86	F_5536_ = 5.342 *p =* 0.0001 R^2^ = 0.047	intercept	F_1536_ = 635.95 (*p* < 0.0001)	0.542	1.000
							Martial arts/control	F_1536_ = 0.01 (*p =* 0.9207)	0.00002	0.051
							*DRD2* rs1799732	F_2536_ = 0.72 (*p =* 0.4880)	0.003	0.171
							Martial arts/control × *DRD2* rs1799732	F_2536_ = 4.25 (*p =* 0.0147)	0.016	0.743
Reward dependence	10.10 ± 3.00	10.39 ± 2.90	10.38 ± 2.89	9.56 ± 3.14	10.75 ± 3.27	F_5536_ = 2.922 *p =* 0.0130 R^2^ = 0.0265	intercept	F_1536_ = 1447.98 (*p* < 0.0001)	0.729	1.000
							Martial arts/control	F_1536_ = 3.95 (*p =* 0.0472)	0.007	0.510
							*DRD2* rs1799732	F_2536_ = 3.10 (*p* = 0.0457)	0.011	0.597
							Martial arts/control × *DRD2* rs1799732	F_2536_ = 3.50 (*p =* 0.0310)	0.013	0.652
Self-directedness	26.72 ± 4.31	23.67 ± 5.07	25.00 ± 4.99	25.52 ± 5.03	26.31 ± 3.99	F_5536_ = 12.10 *p =* 0.0000 R^2^ = 0.101	intercept	F_1536_ = 3439.64 (*p* < 0.0001)	0.865	1.000
							Martial arts/control	F_1536_ = 4.42 (*p =* 0.0358)	0.008	0.556
							*DRD2* rs1799732	F_2536_ = 0.96 (*p =* 0.3834)	0.004	0.217
							Martial arts/control × *DRD2* rs1799732	F_2536_ = 1.21 (*p =* 0.2975)	0.005	0.265
Cooperation	20.59 ± 4.54	19.98 ± 4.63	20.21 ± 4.61	19.99 ± 4.49	23.31 ± 3.74	F_5536_ = 2.314 *p =* 0.0426 R^2^ = 0.021	intercept	F_1536_ = 2522.29 (*p* < 0.0001)	0.825	1.000
							Martial arts/control	F_1536_ = 0.18 (*p =* 0.6714)	0.0003	0.070
							*DRD2* rs1799732	F_2536_ = 3.94 (*p =* 0.0201)	0.0145	0.707
							Martial arts/control × *DRD2* rs1799732	F_2536_ = 0.93 (*p =* 0.3916)	0.003	0.213
Self-transcendence	6.95 ± 3.56	7.04 ± 3.54	7.14 ± 3.54	6.71 ± 3.63	4.68 ± 2.38	F_5536_ = 1.744 *p =* 0.1227 R^2^ = 0.0160	intercept	F_1536_ = 359.72 (*p* < 0.0001)	0.402	1.000
							Martial arts/control	F_1536_ = 0.52 (*p =* 0.4689)	0.001	0.111
							*DRD2* rs1799732	F_2536_ = 3.793 (*p =* 0.0231)	0.013	0.690
							Martial arts/control × *DRD2* rs1799732	F_2536_ = 0.26 (*p =* 0.7718)	0.001	0.091

M—mean, SD—standard deviation. Statistically significant between-group differences are marked in bold print.

## Data Availability

Not applicable.
